# Societal Economic Burden of Cystic Fibrosis in Iran: A Cost-of-Illness Study

**DOI:** 10.36469/001c.143266

**Published:** 2025-09-09

**Authors:** Hassan Karami, Shideh Rafati, Maryam Shirvani Shiri, Ali Mouseli, Hedayat Salari, Amin Ghanbarnejad, Mitra Nowrouzpour, Fatemeh Noroozian, Ali Alizadeh, Fatemeh Asadi, Narges Salehi

**Affiliations:** 1 Social Determinants in Health Promotion Research Center, Hormozgan Health Institute, Hormozgan University of Medical Sciences, Bandar Abbas, Iran; 2 Department of Health Services Management, School of Health, Hormozgan University of Medical Sciences, Bandar Abbas, Iran; 3 Department of Health Policy & Management, School of Health and Nutrition, Bushehr University of Medical Sciences, Bushehr, Iran; 4 National Center for Health Insurance Research, Tehran, Iran; 5 Student Research Committee, Faculty of Nursing and Midwifery, Hormozgan University of Medical Sciences, Bandar Abbas, Iran

**Keywords:** cost of illness, cystic fibrosis, economic burden, health economics, cost analysis

## Abstract

**Background:**

Cystic fibrosis (CF) is a rare genetic disorder that places a substantial financial burden on patients, families, and the healthcare system. This study aimed to estimate the economic impact of CF in southern Iran.

**Methods:**

A cross-sectional, prevalence-based cost-of-illness study was conducted from a societal perspective, using a bottom-up approach and the human capital method. Data were collected through insurance records and a standardized cost questionnaire. Mean annual per-patient costs were calculated, and cost determinants were analyzed using the Mann-Whitney and Kruskal-Wallis tests.

**Results:**

The average annual cost per CF patient was US $4070, with 67% attributed to direct medical costs, 20% to direct nonmedical costs, and 13% to indirect costs. Higher total costs were significantly associated with disease severity, hospitalization history, and absence of supplementary insurance.

**Conclusion:**

CF imposes a considerable economic burden in Iran, predominantly driven by drug and hospitalization expenses. Direct nonmedical costs and indirect costs also contribute meaningfully. These findings highlight the need for improved access to specialized CF care, enhanced insurance coverage, and stronger support for informal caregivers to alleviate the financial pressure on affected families.

## INTRODUCTION

Cystic fibrosis (CF) is one of the most common life-shortening genetic disorders worldwide.^1^ Globally, the prevalence of CF varies by region, ranging from approximately 1 in 3000 to 1 in 6000 live births.^2^ A recent global analysis across 94 countries estimated the number of CF patients at approximately 162 000.^3^ Although Iran currently lacks a national CF patient registry, analyses of CF-specific medication consumption and other indirect indicators suggest that approximately 3000 to 4000 individuals may be affected.^4,5^ Despite the clinical and genetic burden of CF, no comprehensive study has yet examined the economic costs associated with this condition in Iran. The absence of such data poses a major challenge for evidence-informed policymaking and hinders the optimal allocation of resources for rare diseases.

Advances in diagnosis and treatment have significantly increased life expectancy, with many patients now living into their forties and fifties.^6^ However, managing CF requires complex, lifelong, and increasingly intensive interventions, whose scope and cost rise with age, placing a growing financial burden on healthcare systems. In addition, CF imposes considerable indirect and productivity-related costs on families.^7^

Between 1990 and 2022, only 39 studies worldwide have evaluated the costs associated with CF. Notably, fewer than one-third of these studies adopted a comprehensive approach that simultaneously considers the perspectives of healthcare systems, households, and society.^8^ Moreover, the wide range of reported costs—from US $451 to US $160 000—is likely due to differences in methodology, inclusion of indirect costs (IC), patient severity, and access to advanced treatments such as CF transmembrane conductance regulator (CFTR) modulators.^8^

Despite substantial advances in CF treatment in high-income countries, particularly with the introduction of CFTR modulator drugs, the standard care for CF patients in Iran remains primarily supportive, relying on antibiotics, bronchodilators, pancreatic enzymes, vitamin supplements, and respiratory physiotherapy devices.^5^ CFTR modulators and other advanced therapies are not widely accessible in Iran. This limited availability has contributed to poorer clinical outcomes and reduced life expectancy, resulting in a younger CF population with more severe disease compared with countries where these therapies are available.^9,10^ Such disparities complicate international comparisons of CF-related costs and emphasize the importance of region-specific studies to better understand the local economic burden.

Given the significance of this issue, and the current lack of national data, this study aimed to estimate the costs of CF from a societal perspective in Iran and to examine how these costs relate to the clinical and socioeconomic characteristics of patients.

## METHODS

### Study Design

**Figure 1.**
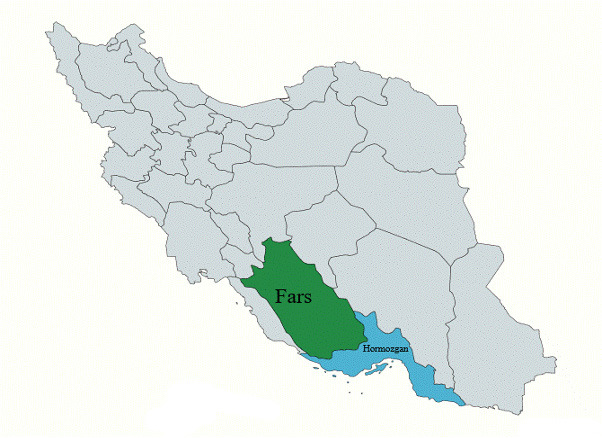
Map of Iran Highlighting the Locations of Fars and Hormozgan Provinces^13^

This retrospective cross-sectional study was conducted between November 2024 and March 2025 in the southern Iranian provinces of Hormozgan and Fars. The entire study period corresponds to the Iranian calendar year 1403, and all cost estimates were calculated based on the official tariffs and economic indicators applicable to that year. According to the 2016 national census, these provinces had a combined population of 6 627 689.^11^ **Figure 1** shows their geographical location within Iran. The study adhered to the Strengthening the Reporting of Observational Studies in Epidemiology (STROBE) guidelines, ensuring methodological rigor and transparency.^12,13^

### Setting, Study Subjects, and Eligibility Criteria

This study focused on CF patients registered with Iran’s two main public insurers—the Social Security Insurance Organization (Tamin Ejtemaei) and the Iranian Health Insurance Organization (Salamat)—which together cover over 90% of the population. Eligible participants were identified through insurer databases, which require verified medical documentation of a CF diagnosis for drug coverage approval. Inclusion criteria included having a valid address and phone number. Individuals with cognitive or physical impairments that hindered participation, as well as those unwilling or unable to take part, were excluded.

### Outcomes, Variables, and Diagnostic Criteria

**Outcomes**: The primary outcome was the average annual total costs (TC) per patient, assessed using a structured questionnaire, data extracted from patients’ insurance records, and insurance billing information.

**Variables**: Sociodemographic and clinical variables—including sex, age, place of residence, education level, marital status, supplementary insurance coverage, body mass index (BMI), disease severity and duration, hospitalization history within the past year, comorbidities, and family history of CF—were analyzed in relation to various cost categories: direct medical costs (DMC), direct nonmedical costs (DNMC), IC, and TC.

**Diagnostic criteria**: Diagnosis of CF was established based on specialist-confirmed documentation submitted for insurance approval, which included at least 1 standard diagnostic test, such as a positive sweat chloride test (above the diagnostic threshold) or CFTR genetic mutation analysis. Perceived disease severity was assessed using a structured self-report instrument; for children under 8 years old, responses were provided by their primary caregivers. The validity of this method has been supported by previous studies showing significant correlations with objective clinical measures, including forced expiratory volume in 1 second, and clinical scoring systems.^14^

### Sample and Sampling

Given the rarity of CF and the need for comprehensive data, a census-based approach was employed instead of sampling. The study encompassed all CF patients covered by the Social Security Insurance Organization (Tamin Ejtemaei) and the Health Insurance Organization (Salamat), thereby minimizing selection bias, enhancing statistical power, and enabling a comprehensive assessment of CF-related costs in the region.

### Costing Methodology

This cost-of-illness study utilized a bottom-up, prevalence-based approach to assess healthcare resource utilization and the economic burden from a societal perspective, including DMC, DNMC, and IC. Data were collected at the individual patient level and, when applicable, from caregivers. The questionnaire recorded resource use over the previous 6 months (12 months for hospitalizations), with 6-month data extrapolated to annual estimates. A 6-month recall period was considered appropriate to ensure accuracy and reliability.^15^ Healthcare expenditures were calculated using national tariffs, recorded in Iranian rials, and converted to US dollars based on the 2025 moving average exchange rate of 570 451 rials per US dollar.^16^

**Determining DMC**: Direct medical costs included physician visits, medications, hospitalizations, imaging, and laboratory tests. For all services except medications, costs were calculated by multiplying the quantity of services by tariffs set annually by the Ministry of Health and Medical Education for both public and private sectors. Given the societal perspective of the study, private sector tariffs were used as a proxy for the actual service costs when estimating DMC. Medication costs were calculated by multiplying the quantity of each drug by its unit price as listed by Iran’s Food and Drug Administration (https://irc.fda.gov.ir/nfi). Expenses related to traditional medicine, such as herbal remedies or traditional manual therapies, were self-reported by patients through the cost questionnaire. These amounts were recorded as reported, without adjustment or imputation based on external price references.

**Determining DNMC**: DNMC included expenses related to medical-related goods, as well as accommodation and transportation costs for patients and their caregivers during visits to healthcare facilities. Medical-related goods comprised items necessary for home-based care and disease management, such as nebulizers, air humidifiers, and disposable medical supplies (eg, syringes, gloves). These items were reported by patients in the cost survey. Transportation costs, as a component of DNMC, were estimated based on the type of transportation used. For patients using public transportation (including buses, subways, trains, airplanes, or traditional/app-based taxis), the cost per visit was calculated using official municipal tariffs or average market fares and then multiplied by the number of visits.^17^ For those using private vehicles, transportation costs were estimated based on the round-trip distance between the patient’s residence and the healthcare facility, applying a standard reimbursement rate per kilometer.^18^ Data on transportation type, number of visits, and round-trip distance were collected via patient-completed questionnaires.

**Determining IC**: The human capital approach was used to estimate IC, with productivity losses due to disability as a major component. Disability was classified into short-term and long-term categories, both included in the overall calculation of IC. For informal care provided by employed caregivers under 65 years of age, costs were estimated based on the average net hourly wage.^19^

Productivity losses due to short-term disability were estimated based on temporary absenteeism from work by both patients and their caregivers. To calculate productivity loss associated with therapy appointments, the hourly wage of the lowest-paid unskilled government worker, as defined by the Ministry of Labour,^20^ was multiplied by the total number of work hours missed by patients and caregivers.

Productivity losses due to long-term disability were assessed by considering early retirement among patients. The cost associated with early retirement was calculated as the difference between the reduced pension received by those retiring early and the full pension they would have been entitled to had they retired at the standard retirement age.

### Data Sources and Measurements

In addition to patient electronic records, data were collected using a custom-designed questionnaire comprising two sections:

**Sociodemographic and clinical history**: This section collected data on patients’ sociodemographic characteristics and clinical history, including sex, place of residence, supplementary insurance coverage, education level, age, marital status, disease severity and duration, hospitalization history within the past year, BMI, and family history of CF.

**Standard cost-of-illness questionnaire**: The questionnaire used in this study was a standardized cost-of-illness instrument adapted from a previous Iranian study^21^ and customized to align with the specific objectives of the current research, focusing on the assessment of both DMC and IC in CF patients. Although the instrument was based on a prior Iranian study, its terminology and phrasing, particularly regarding concepts such as “productivity loss” and “indirect cost,” were reviewed and refined to ensure clarity and cultural relevance for the current target population.

### Data Collection

In collaboration with the Social Security and Health Insurance offices of the studied provinces, a list of CF patients was compiled. After explaining the study objectives and obtaining oral consent, data collection tools along with instructions were provided. The questionnaire link was distributed via social media platforms (Eitaa, WhatsApp, Telegram) to the patients’ registered phone numbers. For those unable to respond, the link was forwarded to a family member, who received guidance on how to complete the questionnaire. Direct medical costs, including physician visits, medications, paraclinical services, and hospitalizations, were obtained from patients’ insurance billing records. The questionnaire also collected information on any DMC not covered by insurance, which was added to the total DMC.

### Ethics Approval and Consent to Participate

The study was approved in 2024 by the Ethics Committee of Hormozgan University of Medical Sciences (IR.HUMS.REC.1403.251). Informed consent was obtained from all participants prior to their inclusion in the study.

### Bias Considerations

To minimize potential biases and enhance the study’s validity, a census-based approach was employed to ensure a representative sample of CF patients, thereby reducing selection bias. To mitigate response bias, multiple follow-ups were conducted, and trained family members assisted participants who were illiterate or who lacked access to social media, encouraging broader participation.

### Handling of Quantitative Variables

Quantitative variables such as age, disease duration, and BMI were initially treated as continuous variables. For descriptive and comparative analyses, these variables were categorized based on sample distribution characteristics: age and disease duration were divided into 2 or 3 groups according to their means and interquartile ranges, while BMI was grouped using clinical thresholds (eg, <15 and ≥15) relevant to the nutritional status of CF patients. These categorizations enhanced interpretability and facilitated subgroup comparisons. No data transformations (eg, logarithmic or spline functions) were applied, as visual inspection of scatter plots and residuals showed no significant deviations from linearity in multivariable models.

### Statistical Analysis

Continuous variables are presented as means with SD, while categorical variables are reported as frequencies and percentages. Cost data are expressed as means with standard errors. The Mann-Whitney U test and Kruskal-Wallis test were used to compare mean costs across groups. Missing data were minimal, accounting for less than 4% of the total data set. Given this low proportion, a complete case analysis approach was adopted, including only fully completed responses in the statistical analysis. This method is considered appropriate when the level of missingness is low and assumed to be missing completely at random. Statistical analyses were performed using IBM SPSS Statistics version 27 (IBM Corp.) and STATA version 17 (StataCorp).

## RESULTS

### Recruitment

**Figure 2.**
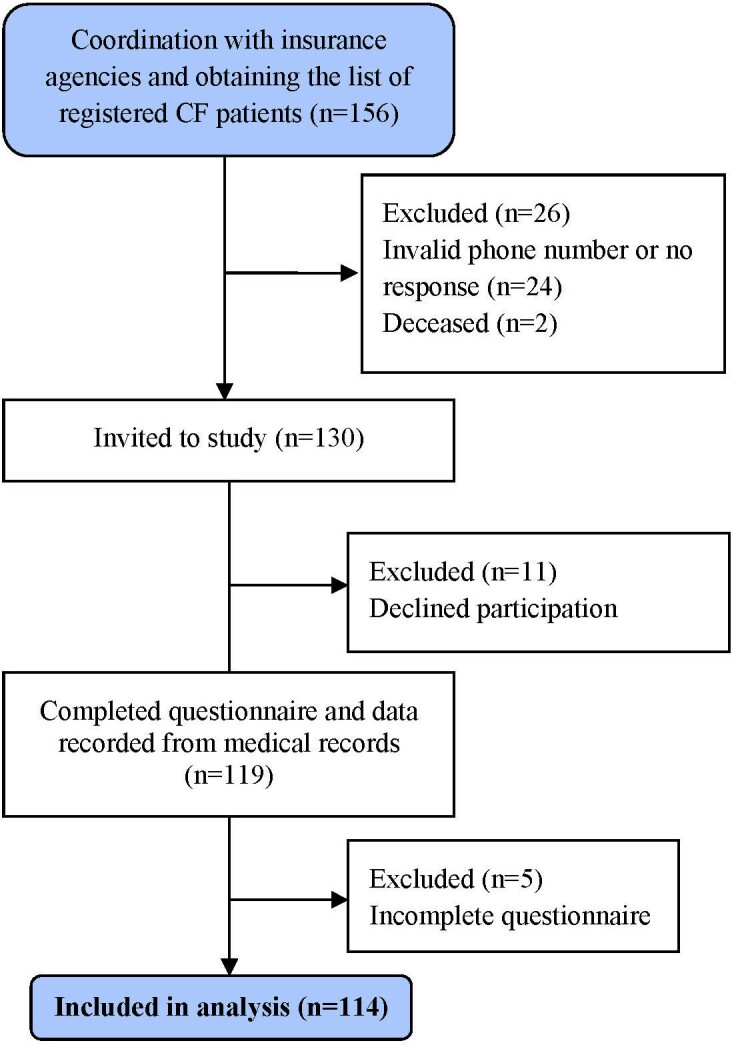
Research Flowchart

A total of 156 eligible participants were identified from insurance records. Among these, 2 had died, 24 were unreachable or had invalid contact information, and 11 declined participation. After data cleaning and handling missing data, 114 patients were included in the final analysis. Of these, 32% were from Hormozgan Province and 68% from Fars Province (**Figure 2**).

### Patient Characteristics

The mean age of patients was 12.6 years, with an average disease duration of 10.4 years. The sample included 54% males, 27% with supplementary insurance, 53% with a history of hospitalization, and disease severity categorized as 48% moderate, 36% severe, and 16% mild. Missing data were minimal (<4%) (**Table 1**).

**Table 1.** Baseline Characteristics of Patients With CF

**Characteristics**	**Mean (SD)**	**N (%)**
Quantitative variables		
Age, y	12.59 (7.33)	
BMI	15.08 (3.71)	
Duration of illness, days	10.36 (5.18)	
Qualitative variables (n=114), n (%)		114 (100)
Gender		62 (54.4)
Male		52 (45.6)
Female		
Age groups		37 (32.5)
<10 y		55 (48.2)
10-15 y		22 (19.3)
>15 y		
BMI		59 (51.8)
<15		55 (48.2)
≥15		
Residence		40 (35.1)
Rural		74 (64.9)
Urban		
Marital status		23 (20.2)
Single		7 (6.1)
Married		84 (73.7)
Patients aged <15 y		62 (54.4)
Education		
<6 grade		43 (37.7)
6-12 grade		41 (36.0)
>12 grade		12 (10.5)
Patients aged <7 y		18 (15.8)
Supplemental insurance		
No		83 (72.8)
Yes		31 (27.2)
Occupation		
Unemployed		19 (16.7)
Employed/student		95 (83.3)
Hospitalization		
No		54 (47.4)
Yes		60 (52.6)
Duration of illness		
<10		57 (50.0)
≥10		57 (50.0)
Family history of disease		
No		93 (81.6)
Yes		21 (18.4)
Disease severity		
Mild		18 (15.8)
Moderate		55 (48.2)
Severe		41 (36.0)

Abbreviations: BMI, body mass index; CF, cystic fibrosis.

### Direct Medical Costs

The mean annual DMC per CF patient was US $2733.64, representing 67.2% of TC. Medications accounted for the largest share (US $1985.08; 48.8%), followed by hospitalization (US $620.06; 15.2%). Other components were considerably smaller: specialist visits (0.9%), laboratory tests (0.5%), general practitioner visits (0.3%), imaging (0.3%), and traditional medicine (1.2%) (**Table 2**).

**Table 2.** Annual DMC, DNMC, and IC of CF per Patient and Resource Utilization (2024 USD)

**Cost Category**	**Participants Using Resources, n (%)**	**Mean (SE), US $**	**Percentage of TC**
General physician	89 (78.1)	12.62 (0.88)	0.31
Specialist physician	112 (98.2)	35.77 (2.23)	0.88
Imaging	76 (66.7)	10.38 (1.08)	0.25
Laboratory tests	92 (80.7)	20.21 (1.46)	0.50
Hospitalization	60 (52.6)	620.06 (99.46)	15.23
Pharmaceuticals	114 (100)	1985.08 (113.80)	48.77
Traditional medicine	22 (19.3)	49.50 (14.49)	1.22
Total DMC	114 (100)	2733.64 (171.80)	67.16
Transportation	111 (97.4)	104.25 (11.61)	2.56
Accommodation	112 (98.2)	370.77 (31.23)	9.11
Home nursing services	8 (7.0)	28.42 (10.38)	0.70
Medical related goods	43 (37.7)	309.80 (93.37)	7.61
Total DNMC	113 (99.1)	813.24 (102.44)	19.98
Patient absenteeism (patients aged ≥15 y)^a^	12 (40)	4.06 (1.50)	0.10
Caregiver absenteeism	109 (95.6)	77.12 (6.90)	1.89
Informal care	89 (78.1)	442.34 (46.44)	10.87
Early retirement (patients aged ≥15 y)^a^	0 (0.0)	0 (0.0)	0.0
Total IC	111 (97.4)	523.52 (47.47)	12.86
Total economic costs	114 (100)	4070.40 (231.62)	100

Abbreviations: CF, cystic fibrosis; DMC, direct medical costs; direct nonmedical costs; IC, indirect costs; TC, total costs; USD, US dollars.

^a^The minimum legal working age in Iran is 15 years and above.

### Direct Nonmedical Costs

The mean annual DNMC per patient was US $813.24, constituting 20% of TC. Accommodation was the largest component (US $370.77; 9.1%), followed by medical-related goods (US $309.80; 7.6%), transportation (2.6%), and home nursing services (0.7%) (**Table 2**).

### Indirect Costs

The mean annual IC per patient was US $523.52, accounting for 12.9% of TC. Informal care constituted 10.9%, caregiver absenteeism 1.9%, and patient absenteeism 0.1% of TC. No costs were reported for early retirement (**Table 2**).

### Total Economic Burden

The average annual TC per patient was US $4070.40, with DMC, DNMC, and IC contributing 67.16%, 19.98%, and 12.86%, respectively, of the TC (**Table 2**).

### Comparison of Economic Costs Among CF Patients

Cost comparisons revealed significant differences: patients with a history of hospitalization had higher DMC and TC; those without supplementary insurance incurred higher DMC, IC, and TC; severe disease was associated with increased DMC, DNMC, and TC; and patients with a BMI ≥15 or living in urban areas had significantly higher IC (**Table 3**).

**Table 3.** Comparison of Economic Costs Among CF Patients (Kruskal-Wallis and Mann-Whitney)

		**Economic Costs (USD), Mean (SE)**		
**Variables**	**DMC**	**DNMC**	**IC**	**TC**
Gender				
Male	2627 (229)	934 (164)	514 (69)	4074 (336)
Female	2861 (260)	670 (109)	535 (65)	4066 (316)
Age groups				
<10 y	2486 (269)	734 (190)	471 (58)	3691 (403)
10-15 y	2794 (264)	743 (115)	485 (67)	4022 (327)
>15 y	2999 (395)	1121 (312)	709 (148)	4829 (551)
BMI				
<15	2875 (257)	956 (180)	436 (54)	4267 (357)
≥15	2582 (227)	660 (86)	618 (78)	3860 (290)
Residence				
Rural	2641 (305)	642 (80)	426 (72)	3709 (332)
Urban	2784 (208)	906 (151)	576 (61)	4266 (307)
Marital status				
Single	3074 (366)	817 (161)	662 (136)	4553 (446)
Married	3193 (858)	1787 (884)	530 (215)	5510 (1108)
Patients aged <15 y	2602 (199)	731 (108)	485 (50)	3818 (271)
Education				
<Grade 6	2800 (296)	815 (193)	551 (73)	4166 (432)
Grade 6-12	2889 (321)	690 (119)	436 (71)	4015 (375)
Grade >12	2340 (205)	1452 (534)	866 (247)	4657 (717)
Patients aged <7 y	2484 (379)	664 (64)	430 (64)	3579 (393)
Supplementary insurance				
No	3032 (214)	811 (118)	582 (60)a	4425 (283)
Yes	1936 (212)	819 (210)	367 (64)	3122 (340)
Occupation				
Unemployed	3285 (520)	1329 (472)	612 (133)	5226 (842)
Employed/student	2623 (177)	710 (77)	506 (51)	3839 (216)
Hospitalization				
No	1945 (175)	640 (84)	546 (71)	3132 (235)
Yes	3443 (254)	969 (178)	503 (64)	4915 (353)
Duration of illness				
<10 days	2777 (244)	754 (137)	536 (65)	4066 (329)
≥10 days	2690 (244)	873 (153)	511 (70)	4074 (329)
Family history of disease				
No	2748 (189)	837 (120)	519 (52)	4104 (265)
Yes	2668 (420)	710 (161)	545 (122)	3923 (467)
Disease severity				
Mild	2394 (435)	417 (89)	440 (77)	3252 (483)
Moderate	2546 (246)	752 (144)	579 (83)	3877 (344)
Severe	3134 (284)	1069 (200)	486 (62)	4689 (381)

Abbreviations: CF, cystic fibrosis; DMC, direct medical costs; direct nonmedical costs; IC, indirect costs; SE, standard error; TC, total costs; USD, US dollars.

## DISCUSSION

To our knowledge, this is the first study assessing the economic burden of CF in Iran. Findings showed that the substantial annual costs were mainly driven by hospitalization and medications. Accommodation and informal care represented the largest portions of DNMC and IC, respectively. Higher costs were significantly associated with prior hospitalization, absence of supplementary insurance, and greater disease severity.

The average annual TC per CF patient was approximately US $4070, predominantly attributed to DMC. Globally, DMC, especially drug and hospitalization expenses, constitute the largest share of CF costs, ranging from US $451 to US $160 000 annually.^8^ Variations across countries likely stem from differences in drug pricing, healthcare infrastructure, and insurance coverage.^8,22^

The mean annual DMC in this study (US $2734) was notably lower than figures reported from countries like the United Kingdom (UK) and Germany,^23^ although direct comparisons are limited by differing standards of care.^5^ In Iran, due to restricted access to CFTR modulators, patients tend to be younger and experience poorer disease control. Care mainly involves supportive treatments such as antibiotics (eg, azithromycin, inhaled tobramycin), mucolytics (dornase alfa), pancreatic enzymes, vitamin supplements, and bronchodilators. Pharmaceuticals (48.77%) and inpatient care (15.23%) accounted for the largest DMC shares, consistent with global trends.^24,25^ However, cost patterns vary internationally; for instance, hospitalization dominates costs in Australia, whereas informal care and medications drive expenses in the UK.^22,24^

Access to advanced diagnostic and therapeutic technologies is a key determinant of CF’s economic burden. CFTR modulators in developed countries have improved lung function, reduced hospitalizations, and enhanced quality of life.^26^ Yet, their high costs limit cost-effectiveness in many health systems,^27,28^ unless prices decrease or financial support is available. Insurance coverage for CFTR modulators can reduce antibiotic use, hospitalizations, and work absenteeism, increasing productivity and potentially lowering both direct and indirect costs. Comprehensive economic evaluations and budget impact analyses by the National Institute of Health Technology Assessment (HTA Iran) are crucial to inform policy decisions.

Specialist visits (0.88%) and laboratory tests (0.50%) contributed minimally to DMC in our study, whereas outpatient services have larger shares in Australia (10%) and the UK (7.9%).^22,24^ Traditional medicine costs (1.22%), unreported in Western studies, reflect local treatment practices. In Malaysia, diagnostic tests represent the largest out-of-pocket DMC share (48.4%) ,^29^ contrasting with our findings where medications dominated (48.77%) and diagnostic tests were minimal (0.5%). These differences likely arise from limited specialized service access and prescribing habits.

Annual DNMC averaged US $813 (20% of TC), with accommodation and medical-related goods comprising the largest shares. The high accommodation cost (9.1%) vs transportation (2.6%) suggests prolonged stays in urban centers for specialized care, especially for rural patients. While similar internationally, many European countries report higher transportation and informal care costs often covered by insurance or social programs.^22,24,30^ The substantial out-of-pocket expenses for medical goods in Iran reflect insurance gaps, and only 7% of patients used home nursing—much lower than in high-income countries.^24^ These differences may result from limited access, inadequate social support, and centralized CF care in major cities.

The annual average IC was US $523 (12.86% of TC), mainly from informal care (10.87%) and caregiver absenteeism (1.89%). In countries like the UK and Canada, informal care can account for up to 44% and absenteeism up to 31% of costs,^22,31^ differences influenced by wages, valuation methods, and social support structures.^8,22^ The low caregiver absenteeism in our sample likely reflects patients’ younger age and limited formal employment. The high reliance on informal care aligns with patterns in the UK and Bulgaria, where most patients depend on family support.^32^ Early retirement costs were zero in our study, contrasting with 9.1% of IC in the UK,^22,33^ likely due to the younger, less employed Iranian CF population.

Patients with severe CF incurred significantly higher DMC, DNMC, and TC than those with mild or moderate disease, consistent with prior studies highlighting increased need for specialized care, hospitalizations, and costly medications with greater severity.^6,24,34,35^ History of hospitalization also correlated with higher costs, confirming hospitalization as a major cost driver—sometimes exceeding 50% of TC.^6,23,34,36^

Lack of supplementary insurance was associated with significantly greater DMC, IC, and TC, consistent with global evidence on insurance’s role in reducing financial burden.^36^ In the United States, patients with limited coverage face higher costs than those with comprehensive plans.^37^ Insurance gaps often delay care access, worsening disease progression and increasing costs.^38^

Policy recommendations include expanding specialized CF clinics to reduce travel and accommodation expenses; establishing a national CF registry to enhance targeted healthcare and financial planning; extending insurance coverage for essential home medical equipment such as nebulizers; improving reimbursement for costly and long-term medications; and introducing social support mechanisms for caregivers, especially parents unable to maintain employment due to caregiving.

### Strengths and Limitations

This study provides the first comprehensive economic burden assessment of CF in Iran, integrating insurance data with self-reported costs to improve DMC estimation. It addresses key evidence gaps and informs policymaking. Analysis of demographic and clinical factors aids identification of high-cost groups and resource prioritization.

Limitations include the cross-sectional design, which limits causal inference and temporal cost trends. Despite using a validated questionnaire, recall bias, particularly for out-of-pocket costs, may remain. The study’s geographic focus on Fars and Hormozgan provinces reflects demographic and cultural diversity but may limit national generalizability.^39,40^

Additionally, costs were not analyzed relative to household income, restricting understanding of financial burden at the household level. Future studies should assess cost burden relative to median household income for a more accurate family impact assessment. Long-term income loss due to disability during working years was not fully considered, nor were costs related to disease complications (neurological disorders, cancers, depression). Further research on these aspects is warranted.

## CONCLUSION

Cystic fibrosis imposes a substantial financial burden on patients and families in Iran, particularly among those with severe disease, hospitalization history, and no supplementary insurance. These findings underscore the need to expand specialized services, improve insurance coverage for essential equipment and medications, and strengthen caregiver support to alleviate this burden.

### Disclosures

The authors declare that this research was conducted independently of any commercial or financial relationships that could be interpreted as potential conflicts of interest.

### Data Source Statement

The data for this study are drawn from a larger research project examining multiple aspects of CF. This article specifically reports findings related to the cost of illness among patients, while results addressing other aspects of the project are presented in separate publications. Each paper focuses on distinct research questions, ensuring no overlap or duplication of data between them.

## Data Availability

The data underlying the findings of this study are available from the corresponding author upon reasonable request.
